# Design, Characterization, and Antibacterial Performance of MAPLE-Deposited Coatings of Magnesium Phosphate-Containing Silver Nanoparticles in Biocompatible Concentrations

**DOI:** 10.3390/ijms23147910

**Published:** 2022-07-18

**Authors:** Denisa Alexandra Florea, Valentina Grumezescu, Alexandra Cătălina Bîrcă, Bogdan Ștefan Vasile, Mihaela Mușat, Cristina Chircov, Miruna S. Stan, Alexandru Mihai Grumezescu, Ecaterina Andronescu, Mariana Carmen Chifiriuc

**Affiliations:** 1 Department of Science and Engineering of Oxide Materials and Nanomaterials, Politehnica University of Bucharest, 011061 Bucharest, Romania; denisa.florea94@yahoo.com (D.A.F.); alexandra.birca@upb.ro (A.C.B.); bogdan.vasile@upb.ro (B.Ș.V.); mihaelamusat6@yahoo.com (M.M.); cristina.chircov@upb.ro (C.C.); agrumezescu@upb.ro (A.M.G.); 2 National Institute for Lasers, Plasma and Radiation Physics, 409 Atomistilor Street, 077125 Magurele, Romania; valentina_grumezescu@yahoo.com; 3 Research Institute of the University of Bucharest (ICUB), University of Bucharest, 050657 Bucharest, Romania; miruna.stan@bio.unibuc.ro; 4 Department of Biochemistry and Molecular Biology, Faculty of Biology, University of Bucharest, 050095 Bucharest, Romania; 5 Academy of Romanian Scientists, Ilfov No. 3, 050044 Bucharest, Romania; carmen.chifiriuc@bio.unibuc.ro; 6 Department of Microbiology, Faculty of Biology, University of Bucharest, Aleea Portocalelor Str. 1-3, District 5, 060101 Bucharest, Romania; 7 The Romanian Academy, Calea Victoriei 25, District 1, 010071 Bucharest, Romania

**Keywords:** silver nanoparticles, magnesium phosphate, MAPLE, biofilms, osteoblasts, *S. aureus*, *Ps. aeruginosa*

## Abstract

Bone disorders and traumas represent a common type of healthcare emergency affecting men and women worldwide. Since most of these diseases imply surgery, frequently complicated by exogenous or endogenous infections, there is an acute need for improving their therapeutic approaches, particularly in clinical conditions requiring orthopedic implants. Various biomaterials have been investigated in the last decades for their potential to increase bone regeneration and prevent orthopedic infections. The present study aimed to develop a series of MAPLE-deposited coatings composed of magnesium phosphate (Mg_3_(PO_4_)_2_) and silver nanoparticles (AgNPs) designed to ensure osteoblast proliferation and anti-infective properties simultaneously. Mg_3_(PO_4_)_2_ and AgNPs were obtained through the cooling bath reaction and chemical reduction, respectively, and then characterized through X-ray Diffraction (XRD), Transmission Electron Microscopy (TEM), and Selected Area Electron Diffraction (SAED). Subsequently, the obtained coatings were evaluated by Infrared Microscopy (IRM), Fourier-Transform Infrared Spectroscopy (FT-IR), and Scanning Electron Microscopy (SEM). Their biological properties show that the proposed composite coatings exhibit well-balanced biocompatibility and antibacterial activity, promoting osteoblasts viability and proliferation and inhibiting the adherence and growth of *Staphylococcus aureus* and *Pseudomonas aeruginosa,* two of the most important agents of orthopedic implant-associated infections.

## 1. Introduction

Bone tissue diseases are a very common type of disorder, affecting men and women worldwide and accounting for half of all chronic conditions in people over 50 years [1,2]. Bone fractures represent both a category or a primary consequence of other bone disorders, occurring especially by incomplete bone healing and/or inefficient bone regeneration [3]. Orthopedic surgery is often inevitable, but unfortunately, it further leads to severe side effects, such as associated infections, nerve injuries, chronic pain, and morbidity. Therefore, developing more efficient solutions in terms of functionality and economic aspects is crucial. Various synthetic and natural biomaterials have been investigated in the last decades to minimize the drawbacks of currently available orthopedic treatments and to reduce the risk of a second surgery due to infection (associated with biofilm formation, bone, and soft tissue necrosis, bone matrix destruction) or other related complications [4]. Biomaterials could exhibit antimicrobial activities *per se* or by providing the local release of antibiotics, modulating the local inflammatory reaction, promoting osteoblast proliferation and bone tissue regeneration, or be used as a bone defect filler after debridement surgery [5].

Among the numerous biomaterials designed for orthopedic applications, ceramics represent promising candidates, providing suitable structures, porosities, mechanical properties, degradation rates, and antimicrobial features. Besides the well-known hydroxyapatite, fluoroapatite, β-tricalcium phosphate, and other bioactive glasses, magnesium phosphate (Mg_3_(PO_4_)_2_) has received increasing attention for orthopedic applications due to its good biocompatibility, suitable mechanical properties, and high biodegradability [6,7,8,9,10].

Thus, magnesium-based bioceramics are used to develop cements, macroporous scaffolds, and coatings [11]. Together with calcium, potassium, and sodium, magnesium ions are among the most abundant ions within the human body, exhibiting multiple functions, e.g., DNA stabilization, enzymatic activation, stimulation of cellular growth, and proliferation [12,13,14]. Furthermore, there are multiple advantages to using magnesium over calcium phosphate in the orthopedic field, such as the capacity of magnesium to replace calcium in the bone structure due to their chemical similarity [15]; magnesium ions assure a better stimulation of native bone formation [16,17]; higher dissolution rates and thus higher biodegradability of magnesium phosphates [13]; magnesium ions prevent the hydroxyapatite undesired crystallization [18]. Despite these multiple advantages of using magnesium phosphate for bone-related applications, the research is still limited.

Several types of nanoparticles and nanocarriers have been validated as suitable options for limiting infection occurrence, including antibiotic-resistant ones [19,20,21,22,23,24]. Due to their well-known and long use as antimicrobial potential and unique combination of magnetic, optic, and electric properties, silver nanoparticles (AgNPs) have become very popular in the biomedical field [25]. AgNPs have many advantages over conventional antibiotics, making them a promising choice for obtaining bioactive coatings able to release locally antimicrobial species. AgNPs have good stability in the physiological environment, they exhibit microbicidal effects on a broad spectrum of microbial species, and due to their multiple molecular targets, the risk for selecting resistance is low [18]. By killing the microbial cells from the implanted material surrounding tissues and thus, inhibiting the microbial adherence and biofilm development, these coatings will minimize the risk of implant-associated infections [26,27,28,29]. They can be synthesized using various chemical, biological, electrochemical, or ‘green’ methods [26,27,30,31,32,33]. The AgNPs can be deposited on the implant surface by different approaches, aiming to achieve an acceptable balance between their cytotoxicity and antimicrobial effect [34].

Loading of AgNPs into the calcium or magnesium ceramics porous structure has been proposed to obtain biomaterials or implant coatings with high biocompatibility and antibacterial properties [35,36].

We report here the combined use of magnesium phosphate and AgNPs for developing nanostructured functional coatings with anti-adherent properties to avoid bacterial cell adhesion, spread, and growth, and to simultaneously stimulate bone regeneration. We have used the Matrix-Assisted Pulsed Laser Evaporation (MAPLE) technique to deposit the bioactive thin films on the implant material surface for obtaining homogenous coatings.

The AgNPs were obtained by chemical reduction, while Mg_3_(PO_4_)_2_ was synthesized by the cooling bath reaction, as both methods are characterized by high efficiencies and reaction yields [37]. The MAPLE technique allowed us to control the properties of the coatings in terms of size, rugosity, and thickness, which makes them suitable for implant coating development [38,39]. The presence of AgNPs provided the obtained coatings with antimicrobial properties, while Mg_3_(PO_4_)_2_ contributed its good biocompatibility, high biodegradability rate, and the capacity of interaction with osteoblasts that will lead to a faster implant integration.

## 2. Results and Discussion

The present study aimed to develop nanostructured coatings for orthopedic implant applications that would concomitantly ensure a bone regenerative potential through the presence of Mg_3_(PO_4_)_2_ and an antimicrobial character by adding AgNPs. There are many studies focused on magnetite [40,41,42,43,44,45,46], hydroxyapatite [47,48,49,50], and silver [51,52] nanoparticles for coatings obtained by MAPLE technique; no available studies report the combined use of AgNPs and Mg_3_(PO_4_)_2_. The characterization of the biomaterials involved the physicochemical analyses of AgNPs and Mg_3_(PO_4_)_2_ and the physicochemical, biological, and antimicrobial evaluation of the obtained coatings.

### 2.1. Physicochemical Characterization of AgNPs

**Figure 1:**
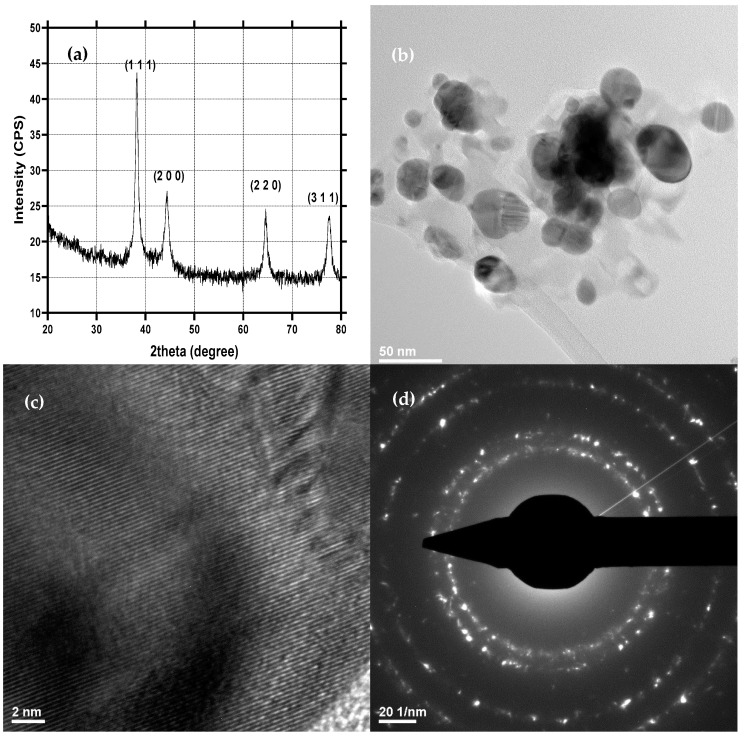

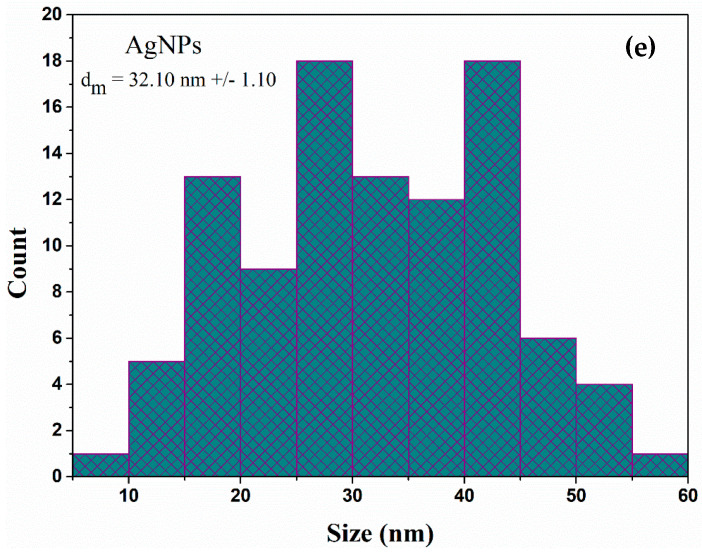
XRD spectrum (**a**), TEM and HR-TEM micrographs (**b**,**c**), SAED patterns (**d**) and size distribution (**e**) of AgNPs.

AgNPs were evaluated through the XRD, TEM, and SAED techniques (Figure 1). The acquired diffractogram demonstrated the formation of silver as a unique crystalline phase in the Fm-3m cubic crystal system, as shown by the associated Miller indices (JCPDS 04-014-0266 [53]) (Figure 1a). The diffraction peaks are narrow, indicating a relatively large crystallite size. The SAED patterns further confirmed the results, as the diffraction rings correspond to the Miller indices specific for silver in the cubic system (Figure 1d). Furthermore, the crystallinity of the obtained AgNPs is demonstrated through the high intensity of the diffraction peaks within the diffractograms and by the non-diffused diffraction rings in the SAED characteristic of highly crystalline materials [54]. As can be seen in the TEM micrographs, the nanoparticles are quasispherical with an average diameter of 32.1 ± 1.1 nm and an increased tendency for agglomeration (Figure 1b).

### 2.2. Physicochemical Characterization of Mg_3_(PO_4_)_2_

**Figure 2:**
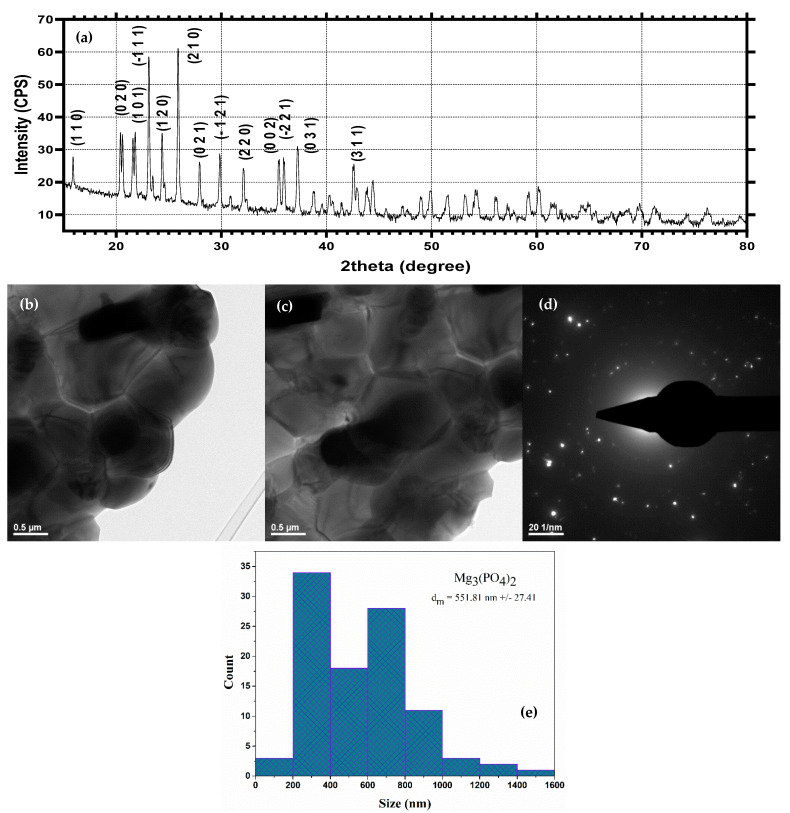
XRD spectrum (**a**), TEM micrographs (**b**,**c**), SAED patterns (**d**) and size distribution (**e**) of Mg_3_(PO_4_)_2_.

Similarly, Mg_3_(PO_4_)_2_ was characterized using the XRD, TEM, and SAED techniques (Figure 2). The diffractogram confirms the formation of a highly crystalline Mg_3_(PO_4_)_2_ in the P21/n monoclinic system through the specific diffraction peaks and the associated Miller indices (JCPDS 00-033-0876 [55,56]) (Figure 2a). The SAED patterns reveal the presence of spots, which are generally characteristic of monocrystalline materials (Figure 2d). Thus, it can be stated that the Mg_3_(PO_4_)_2_ particles are monocrystalline, with the crystallite size corresponding to the particle size. Furthermore, TEM images reveal the formation of polyhedral particles with a uniform size of 551.81 ± 27.41 nm (Figure 2b,c).

### 2.3. Physicochemical Characterization of the AgNPs/Mg_3_(PO_4_)_2_ Coatings

The integrity of the functional groups and the composition after MAPLE deposition of the AgNPs/Mg_3_(PO_4_)_2_ coatings were evaluated through IR mapping. As a reference material, the drop-cast was used. The IR maps were created using the second derivative minimum of the spectral data [57]. The absorption peaks used as spectral markers are characteristic of the stretching of the P-O bond within the phosphate group at around 1100 and 800 cm^−1^ [58,59].

**Figure 3:**
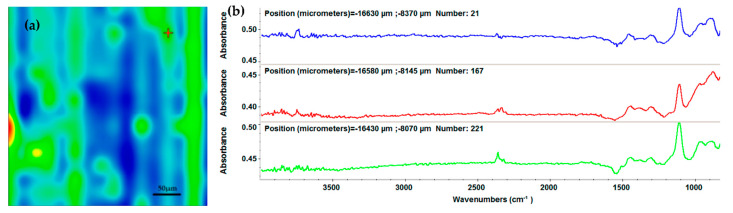
IR map (**a**) and FT-IR spectra (**b**) of AgNPs/Mg_3_(PO_4_)_2_ drop-cast.

**Figure 4:**
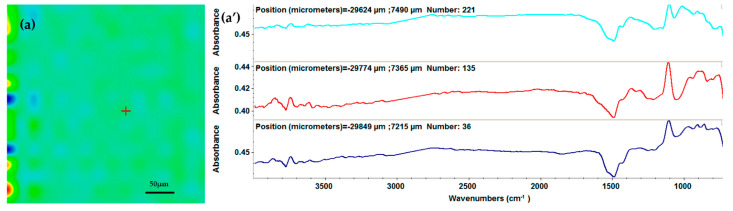

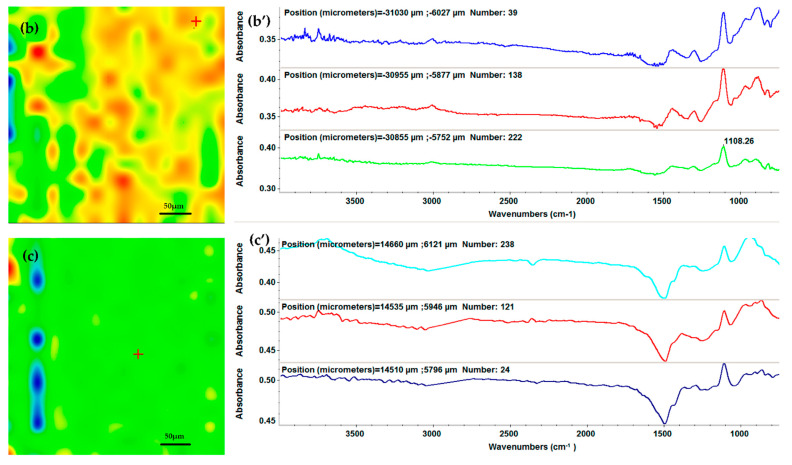
IR maps (**a**–**c**) and FT-IR spectra (**a’**–**c’**) of AgNPs/Mg_3_(PO_4_)_2_ coatings obtained by MAPLE tehnicque at 200 mJ/cm^2^ (**a**,**a’**), 300 mJ/cm^2^ (**b**,**b’**), and 400 mJ/cm^2^ (**c**,**c’**) laser fluences.

In this context, Figure 3a shows the IR micrographs of the AgNPs/Mg_3_(PO_4_)_2_ obtained through drop-cast. As it can be seen, the IR map shows multiple color zones, which are attributed to the amount of the AgNPs/Mg_3_(PO_4_)_2_ material present. As the acquired results demonstrate, the drop-cast does not provide a uniform substrate coating. However, the FT-IR results from Figure 3b can be used as reference spectra in order to assess the integrity of the functional groups. FT-IR spectra (Figure 3b) confirm the presence of PO_4_^3−^ at 1108 cm^−1^. This peak was further used to create the IR maps by monitoring its intensity. In this context, the IR mapping and FT-IR results of the coatings at three different laser fluences are depicted in Figure 4.

Furthermore, considering the complementary results from the FT-IR spectra (Figure 4a’–c’), the 200 and 300 mJ/cm^2^ laser fluences do not affect the integrity of the AgNPs/Mg_3_(PO_4_)_2_ coating chemical structures, the latter resulting in higher intensities of the absorption peaks. However, the 400 mJ/cm^2^ fluence led to a noticeable decrease in the absorption peak, which is associated with changes or damages to the material composition by degradation of the functional groups. Therefore, considering the obtained information, the optimal choice for further investigations is the samples processed at 300 mJ/cm^2^ laser fluence.

**Figure 5:**
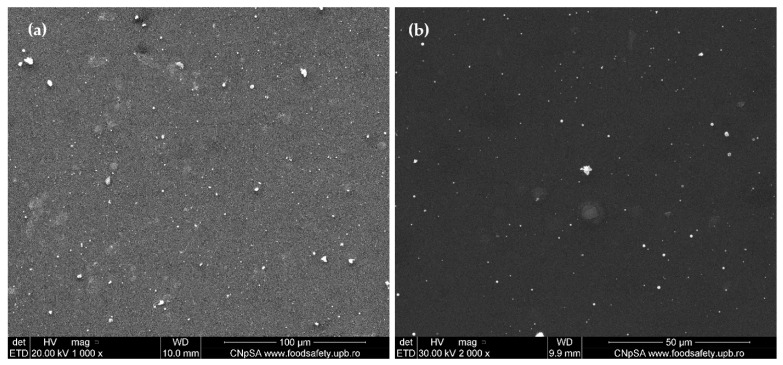

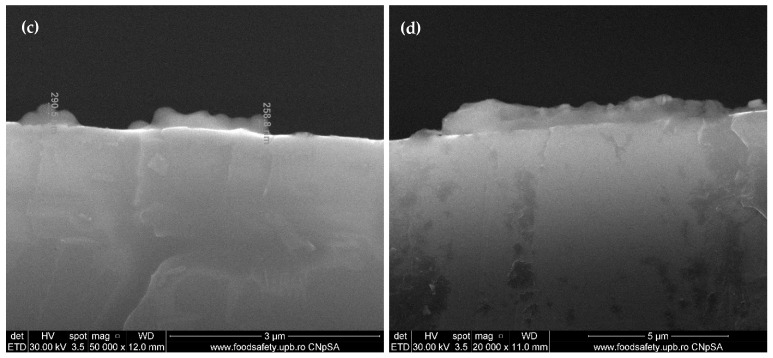
Top-view SEM images (**a**,**b**) and Cross-section SEM images (**c**,**d**) of the AgNPs/Mg_3_(PO_4_)_2_ coatings at different magnifications.

As can be seen from the obtained SEM images (Figure 5), the AgNPs/Mg_3_(PO_4_)_2_ coatings were uniformly deposited onto the titanium substrates. However, the top-view images (Figure 5a,b) at higher magnifications show the presence of agglomerates. The cross-section SEM images confirm the presence of agglomerates within the coatings, varying sizes from 55 nm to 300 nm. The results confirm the IR mappings, as the agglomerates present on the substrate correspond to the red-color areas from the micrographs [41,57,60].

### 2.4. In Vitro Evaluation

#### 2.4.1. Osteoblasts Behavior

**Figure 6:**
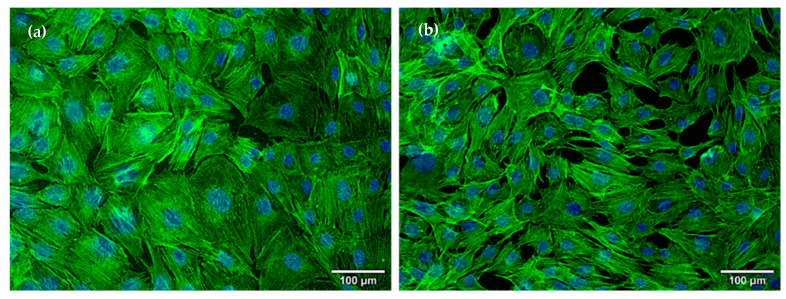
Fluorescence microscopy images for actin filaments (green) and nuclei (blue) staining in MC3T3-E1 osteoblasts grown for 24 h on the surface of control (**a**), and AgNPs/Mg_3_(PO_4_)_2_ (**b**) coatings.

The morphological modifications of MC3T3-E1 osteoblasts in contact with the coatings regarding cytoskeleton organization were evaluated using fluorescence microscopy (Figure 6). The obtained results showed a positive influence of the applied coatings in terms of osteoblast layer confluency and morphology, as the cells present well-organized actin filaments and architecture specific for healthy osteoblasts as also visualized in control. It can be highlighted that these coatings were able to stimulate osteoblasts proliferation, having good biocompatibility and thus an important potential for orthopedic applications, most probably grace to Mg_3_(PO_4_)_2_ presence in the composition.

**Figure 7:**
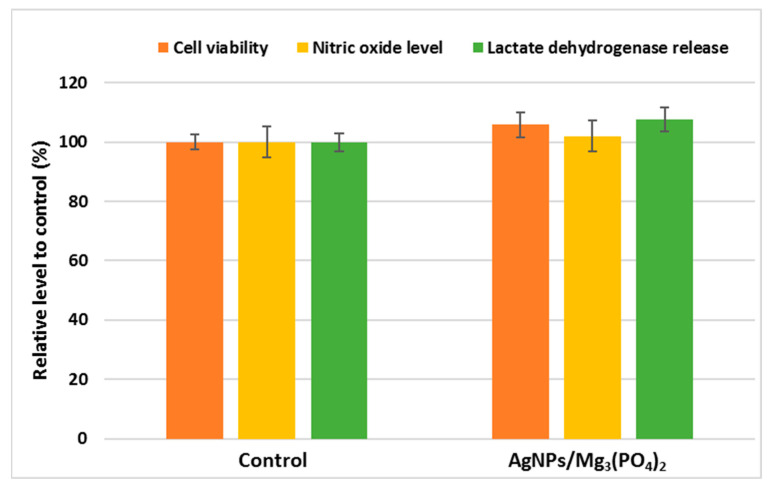
Cell viability, NO level, and LDH release after 24-h growth of MC3T3-E1 osteoblasts on the surface of control and AgNPs/Mg_3_(PO_4_)_2_ coatings.

The MTT assay was used to examine the viability of cells grown on the surface of tested coatings (Figure 7). The obtained results suggested a high survival rate of osteoblasts seeded on the coatings, the levels of cellular viability being higher by 5% compared to control. Furthermore, no significant changes were recorded for the cells grown on the AgNPs/Mg_3_(PO_4_)_2_ sample, in the case of NO level and LDH release, compared to control levels (Figure 7. These results are promising, considering that recent studies show that even in low-dose, AgNPs in suspension could affect the MG-63 cells and thus interfere with bone formation [61]. Moreover, it has been shown that AgNPs exhibit their antibacterial effects at concentrations 2-4 times higher than those inducing cytotoxic effects. Generally, the cytotoxic effects against osteoblasts are directly proportional to the release of Mg ions. They occur at high concentrations, suggesting the existence of a therapeutical window for the application of AgNPs in orthopedic coatings. Still, however, a careful weighing of the anti-infective effects and good biocompatibility is needed [62,63]. One must assure by in vitro studies that the AgNPs concentrations showing antimicrobial activity are lower than the cytotoxic ones to advance with the development of useful tools to fight orthopedic implants associated infections [64]. In the coatings, the released cytotoxic species are probably below the cytotoxic concentration. Therefore, it can be concluded that the AgNPs/Mg_3_(PO_4_)_2_ coatings do not exhibit cytotoxicity in contact with osteoblasts and might lead to faster and uniform bone regeneration in orthopedic applications.

#### 2.4.2. Antimicrobial Activity

Orthopedic surgery is often complicated by the occurrence of post-operative infections produced by *S. aureus*, other Gram-positive organisms, Gram-negative organisms including *P. aeruginosa,* and fungal infections, which, due to their high frequency and antimicrobial resistance features are associated with increased morbidity and mortality, decreased quality of life, and higher hospitalization costs [65,66,67]. Despite the huge technological and medical progress, the incidence of implant-associated infections is increasing proportionally to the number of patients needing implant devices [68]. A very recent retrospective study assessing the 180-day incidence of *S. aureus* infections in adult patients undergoing orthopedic surgery between 2010 and 2015 has shown that among the Gram-positive bacterial strains, *S. aureus* remains the most common pathogen isolated from culture-confirmed surgical site infections (48.0%) and bloodstream infections (35.0%), followed at a distance by Enterobacteriaceae (14.0%). This suggests that *S. aureus* infections continue to be an important contributor to the incidence and burden of orthopedic surgeries [69].

Although the Gram-negative bacteria are isolated with a lower frequency from orthopedic implants associated infections, they remain in the research focus due to their high resistance to currently available antimicrobials, leading to complicated, persistent infections requiring longer hospitalization and higher costs, especially in case of immunocompromised patients [70]. Among the Gram-negative bacterial species, *P. aeruginosa* is considered one of the most fearful and difficult to treat because of its multiple drug resistance and ability to develop biofilms and persist in different environments [71].

Our study aimed to obtain coatings consisting of AgNPs and Mg3(PO4)2 on titanium surfaces by MAPLE technique. As far as we know, the research conducted related to the decoration of magnesium phosphate with AgNPs is still scarce [36].

**Figure 8:**
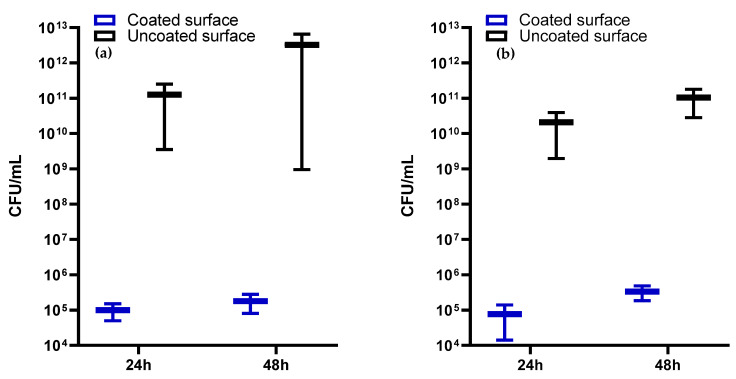
Antibacterial efficiency assessment of the AgNPs/Mg_3_(PO_4_)_2_ coatings against *S. aureus* (**a**) and *P. aeruginosa* (**b**) at 24 and 48 h.

Our models for evaluating the antimicrobial potential of the obtained coatings were represented by *S. aureus* and *P. aeruginosa* strains, which were incubated in the presence of the samples for 48 h. The antibacterial effects of the coatings were quantified after 24 h and 48 h of incubation by assessing the number of viable cell counts. A drastic inhibition of the bacterial growth was recorded for the samples compared to controls, represented by the bare glass (Figure 8). If for the controls, the number of viable cell counts reached ~1 × 10^11^ and ~1 × 10^13^ CFU/mL at 24 h and 48 h, respectively, in the presence of AgNPs/Mg_3_(PO_4_)_2_ coatings considerably decreased values of ~1 × 10^5^ CFU/mL were recorded after 24 h of incubation and maintained until 48 h.

This strong antibacterial effect could be mediated by two mechanisms, i.e., direct contact killing and Ag ion-mediated killing [29].

The direct killing is caused by the adhesion of AgNPs to the bacterial wall and eventually by the penetration of the cellular membrane by the smaller size NPs, where they can interact directly with different intracellular targets or affect them indirectly by stimulating the release of reactive oxygen species (ROS), leading to structural and physiological alterations, incompatible with the microbial cell viability. The release of silver ions from the coatings leads to their interaction mainly with membrane proteins, resulting in their inactivation, but also with nucleic acids, inhibiting DNA replication and, thus, cellular multiplication [72]. These two mechanisms could act synergistically in the cause of our functional coatings.

Despite literature data showing that AgNPs exhibit a stronger bactericidal effect against Gram-negative bacteria, due to their thinner peptidoglycan layer and the presence of a negatively charged outer membrane, favoring the interaction with the positively charged Ag ions [73], no significant differences were observed in the efficacy of the obtained coatings against the two bacterial strains, thus proving the antibacterial effect of the coatings against Gram-positive and Gram-negative bacteria.

Thus, by corroborating the in vitro cytocompatibility and antimicrobial assays results, we could state that the proposed materials exhibit a well-balanced antibacterial effect and biocompatibility, showing promising potential for orthopedic applications.

## 3. Materials and Methods

### 3.1. Materials

Silver nitrate (AgNO_3_), D-glucose (C₆H₁₂O₆), magnesium chloride hexahydrate (MgCl_2_·6H_2_O), phosphoric acid (H_3_PO_4_), sodium hydroxide (NaOH), and dimethyl sulfoxide (99%, DMSO) were purchased from Sigma-Aldrich Merck (Darmstadt, Germany). All chemicals were of analytical purity and used with no further purification.

### 3.2. Synthesis Methods

#### 3.2.1. AgNPs Synthesis

AgNPs were prepared by chemical reduction. 0.5 g of AgNO_3_ was dissolved in 100 mL of deionized water. The solution containing the reduction agent was prepared by dissolving 1 g of D-glucose and 4 g of NaOH in 100 mL of deionized water under magnetic stirring at 80 °C. The second solution was added dropwise in the AgNO_3_ solution, which resulted in a homogenous final product that was filtered and washed with deionized water. Subsequently, the obtained product was dried.

#### 3.2.2. Mg_3_(PO_4_)_2_ Synthesis

The synthesis of Mg_3_(PO_4_)_2_ involved the preparation of two solutions by dissolving MgCl_2_·6H_2_O and H_3_PO_4_ in 200 mL of deionized water. The H_3_PO_4_ solution was added dropwise in the MgCl_2_·6H_2_O, and the pH was adjusted from 2 to 9 using NaOH. The obtained dispersion was subjected to a cooling bath reaction for 24 h. Afterward, the obtained gel was filtered, washed, and dried at room temperature, followed by calcination at 850 °C for 6 h.

#### 3.2.3. Coatings Fabrication by MAPLE Technique

MAPLE targets were prepared by freezing at liquid nitrogen temperature a solution of 4% (*w/v*) AgNPs and Mg_3_(PO_4_)_2_ (1:3 wt%)in DMSO. A KrF* (λ = 248 nm, τFWHM = 25 ns) (COMPexPro 205 Lambda Physics-Coherent) excimer laser beam impinged the target at a laser fluence of 200–400 mJ/cm^2^, the repetition rate of 20 Hz and for 79,000–138,000 pulses. During the laser irradiation, the cryogenic target was maintained at a temperature of ∼173 K by constant cooling with liquid nitrogen, rotated at a rate of 0.4 Hz, and upheld at a 4 cm distance from substrates. All depositions on the titanium disks were conducted at room temperature and a background pressure of 10^−2^ mbar.

### 3.3. Characterization Methods

X-ray Diffraction (XRD). AgNPs and Mg_3_(PO_4_)_2_ were subjected to the XRD analysis using a PANalytical Empyrean diffractometer (PANalytical, Almelo, The Netherlands) provided with CuK_α_ radiation (λ = 1.541874 Å), a hybrid 2 × Ge (220) monochromator for Cu, and a parallel plate collimator on the PIXcel3D detector. Experimental data were acquired between the 2θ angles of 10-80° (incidence angle of 0.5°, step size of 0.04°, and time step of 3 s). The results were processed using the HighScore Plus software (version 3.0, PANalytical, Almelo, The Netherlands).

Transmission Electron Microscopy (TEM). Selected Area Electron Diffraction (SAED). Samples were dispersed into deionized water using an ultrasonic bath, and 10 µL of each suspension was placed on a 400-mesh lacey carbon-coated copper grid at room temperature. TEM micrographs and SAED patterns were obtained using a High-Resolution 80–200 TITAN THEMIS transmission microscope (purchased from FEI, Hillsboro, OR, USA) equipped with an Image Corrector and EDXS detector in the column that is operated at 200 kV in transmission mode.

Scanning Electron Microscopy (SEM). An Inspect F50 high-resolution scanning electron microscope (Thermo Fisher—former FEI, Eindhoven, The Netherlands) was used to assess the morphology and size of the samples at an energy of 30 keV.

Infrared Microscopy (IRM). IRM was performed with a Nicolet iN10 MX FT-IR microscope with an MCT liquid nitrogen cooled detector. The spectral collection was made in the 4000–800 cm^−1^ range at a resolution of 8 cm^−1^ in reflection mode. A total of 32 scans were co-added and converted to absorbance for each spectrum using OmincPicta software (Thermo Scientific, Waltham, MA, USA).

In vitro testing of coatings interaction with osteoblasts cells. Murine osteoblast cells (MC3T3-E1 cell line from American Type Cell Culture) were used to assess the in vitro performance of the coatings. Before testing, all samples were subjected to UV sterilization for 1 h. To determine the cellular viability, the cells were seeded on the coatings at a density of 5 × 10^4^ cells/cm^2^ and kept in the incubator at 37 °C in a humid atmosphere with 5% CO_2_. Glass slides without nanocoating on the surface were used as control. After that, the cell culture medium was taken for the Griess assay to measure the nitric oxide (NO) level and for the lactate dehydrogenase (LDH) release assay. Firstly, the Griess reagent, represented by a stoichiometric solution (*v*/*v*) of 0.1% naphthyl ethylenediamine dihydrochloride and 1% sulphanilamide in 5% H_3_PO_4_, was mixed in equal volumes with the cell culture supernatants. The absorbance of the mix was read at 550 nm using the Tecan Genios microplate reader. Secondly, the amount of released LDH was measured with a cytotoxicity detection kit from Roche, reading the absorbance of the final mix at 450 nm.

The attached cells that remained after medium removal were incubated for 2 h with (3-(4,5-dimethylthiazol-2-yl)-2,5-diphenyltetrazolium bromide solution (1 mg/mL prepared in PBS). The formazan crystals were solubilized with isopropanol, and the absorbance was spectrophotometrically measured at the 595 nm wavelength using a Tecan Genios microplate reader.

Moreover, an Olympus IX71 fluorescence inverted microscope was utilized to evaluate the actin filaments stained with phalloidin-fluorescein isothiocyanate (phalloidin-FITC) after a fixation step with 4% paraformaldehyde and a permeabilization step with 0.5% Triton X-100 and 1% bovine serum albumin. Nuclei were counterstained with 4’,6-diamidino-2-phenylindole (DAPI).

Antimicrobial Performance. The obtained coatings were evaluated in terms of antimicrobial performance and biofilm inhibition. All samples were sterilized using UV exposure for 20 min on each side. The bacterial species used for the antimicrobial evaluation were *S. aureus* and *Ps. aeruginosa.* A cellular suspension was obtained according to specific protocols to quantify the number of units that can form bacterial colonies (CFU/mL) [74].

## 4. Conclusions

The present study aimed to develop a series of biocompatible and antibacterial coatings that can be used in orthopedic applications. The investigated materials consisted of AgNPs and Mg_3_(PO_4_)_2,_ which were synthesized through well-known routes and combined for obtaining nanostructured coatings by the MAPLE technique. Our study demonstrated the efficiency of the AgNPs/Mg_3_(PO_4_)_2_ coatings’ efficiency in promoting osteoblast viability and proliferation while exhibiting antibacterial activity against two of the most important etiological agents of orthopedic implants associated infections. Specifically, the potential of the proposed materials resides in the synergistic effects of Mg_3_(PO_4_)_2_ to ensure bone regeneration and implant integration, while AgNPs provide the antibacterial properties of the composite materials. 

## Data Availability

Not applicable.
